# Prevalence and key radiographic spinal malalignment parameters that influence the risk for gastroesophageal reflux disease in patients treated surgically for adult spinal deformity

**DOI:** 10.1186/s12876-018-0738-6

**Published:** 2018-01-10

**Authors:** Tetsuro Ohba, Shigeto Ebata, Kensuke Koyama, Hirotaka Haro

**Affiliations:** Department of Orthopaedics, University of Yamanashi, 1110, Shimokato, Chuo, Yamanashi, 409-3898 Japan

**Keywords:** Adult spinal deformity, Gastroesophageal reflux disease, Thoracolumbar kyphosis, Fulcrum backward-bending position, Surgical spinal correction, Surgical planning

## Abstract

**Background:**

Gastroesophageal reflux disease (GERD) is a factor that has a significant negative impact on the quality of life (QoL). Vertebral fractures and/or spinal malalignment may influence the frequency of GERD. However, the epidemiology and pathology of GERD in patients with adult spinal deformity (ASD) are still largely unknown. To establish the optimal surgical strategy for GERD in patients treated surgically for ASD, we sought to clarify the GERD prevalence, determine radiographically which spinal malalignment parameters influence GERD risk, and evaluate GERD improvement postoperatively.

**Methods:**

Seventy-one consecutive patients with ASD who were treated with thoracolumbar corrective surgery and followed up for at least 1 year were enrolled. GERD was diagnosed by a gastroenterologist based on proton pump inhibitor medication response and/or an FSSG score > 8 points. Full-length lateral radiographs in a standing posture and in a supine, fulcrum backward-bending (FBB) position were taken preoperatively and 1 year postoperatively, and radiographic parameters were obtained. Correlations between radiographic parameters and FSSG scores were determined by Pearson’s correlation coefficient. Multivariate logistic regression analyses were performed to evaluate the odds ratio (OR) with a 95% confidence interval (95% CI) for potential risk factors for GERD.

**Results:**

Patients were classified into two groups based on GERD symptoms, with 37 (52%) in the GERD+ group. Thoracolumbar kyphosis (TLK) in the FBB position was significantly more common in the GERD+ versus the GERD− group. Multivariate logistic regression analysis showed that lumbar lordosis (LL) and TLK curve in the FBB position significantly influenced the presence of GERD. Other factors showed no association with GERD. Significant improvements in FSSG scores were noted 1 year postoperatively. However, 20 (28.2%) patients still had GERD symptoms. The postoperative TLK curve was highly significantly correlated with FSSG scores 1 year postsurgery.

**Conclusions:**

Of the 71 patients treated surgically for ASD, 37 (52%) had a high frequency of GERD symptoms. An inflexible thoracolumbar curve with increased TLK in the FBB position was significantly associated with GERD symptoms. Despite significant improvements in FSSG scores postoperatively, insufficient correction of TLK might be a risk factor for persistent GERD symptoms.

## Background

In an aging society, adult spinal deformity (ASD) remains one of the most controversial topics within the field of spinal disorders due to the significant impact it can have on health-related quality of life (HRQoL). Spinal malalignment in the sagittal plane has been correlated with pain as well as physical and mental disability [1, 2] [3, 4]. Therefore, numerous studies have attempted to establish mathematical prediction formulas for optimal surgical planning in the treatment of ASD. Attention has been paid to the correlation between radiographic spinopelvic parameters and HRQoL scores evaluated by questionnaires such as the Oswestry Disability Index (ODI), the Scoliosis Research Society-22r, and the Short Form [SF]-12 [4–7].

Gastroesophageal reflux disease (GERD) is recognized as a factor that has a significant negative impact on quality of life (QoL) [8], and a recent large survey showed that 37.6% of Japanese individuals were affected by GERD [9]. In addition, it has been suggested that vertebral fractures and/or spinal malalignment may influence the frequency of GERD [10–13]. However, there are few studies about the epidemiology and pathology of GERD in patients with severe spinal deformity who require treatment with thoracolumbar corrective surgery. Additionally, the efficacy of surgical spinal correction for GERD is still largely unknown. To establish the optimal surgical strategy for treating GERD in patients with ASD, we set out to do the following: 1) to clarify the prevalence of GERD; 2) to determine radiographically which spinal malalignment parameters predominantly influence the risk for GERD; and 3) to evaluate the improvement of GERD after surgical spinal correction.

## Methods

### Patients and surgical techniques

All patients were considered candidates for thoracolumbar correction if a fusion was indicated because of ASD and if a full course of conservative care had been exhausted. The inclusion criteria were age greater than 60 years and a radiographic diagnosis of ASD defined by at least one of the following parameters: a coronal Cobb angle more than 30°; a C7 sagittal vertical axis (SVA), which is the distance between the C7 plumb line and the posterosuperior edge of S1, more than 5 cm; pelvic tilt (PT), which is the orientation of the pelvis with respect to the femurs and the rest of the body, more than 25°; and/or thoracic kyphosis (TK) more than 30°. Patients were excluded if they had inflammatory arthritis, tumors, or neuromuscular diseases. The following information was obtained from each subject: number of vertebral fractures; radiographic parameters (as described below); intake of nonsteroidal anti-inflammatory drugs (NSAIDs), and intake of bisphosphonates. The demographic details of the patients are shown in Table 1.

**Table 1:** Baseline characteristics of patients with ASD with and without GERD

Variable	Overall (*n* = 71)	GERD+ (*n* = 37)	GERD− (*n* = 34)	*P* value
Age (y)	70.4 ± 7.4	70.8 ± 6.54	70.0 ± 8.23	0.6663
Female/male gender (n)	62/9	32/5	30/4	0.7297
TK, standing (°)	24.9 ± 19.0	29.3 ± 19.8	20.7 ± 17.3	0.0620
TLK, standing (°)	19.34 ± 18.4	23.7 ± 19.7	15.2 ± 16.1	0.0561
LL, standing (°)	13.2 ± 19.6	14.4 ± 18.2	12.2 ± 21.7	0.6571
PI (°)	53.1 ± 9.6	54.4 ± 11.2	51.9 ± 8.1	0.2963
PT (°)	37.5 ± 10.6	38.4 ± 10.0	36.5 ± 11.2	0.4759
SS (°)	17.0 ± 11.9	16.9 ± 10.1	17.2 ± 13.4	0.8947
SVA (mm)	104.8 ± 64.6	116.9 ± 56.8	93.1 ± 71.0	0.1354
PI-LL(°)	29.3 ± 16.5	27.52 ± 17.3	31.0 ± 15.4	0.3864
Cobb angle (°)	23.6 ± 16.9	26.9 ± 17.9	20.1 ± 15.1	0.0918
TK, FBB (°)	19.9 ± 15.8	24.2 ± 16.4	14.8 ± 13.6	**<0.05**
TLK, FBB (°)	7.2 ± 14.4	13.6 ± 15.2	−0.42 ± 8.8	**<0.0001**
LL, FBB (°)	31.9 ± 16.3	29.9 ± 15.8	34.3 ± 17.0	0.0918
Cobb angle ~30°/30°~ (n)		22/15	29/5	**<0.05**
Number of thoracolumbar vertebral fractures (n)	0.55 ± 1.1	0.78 ± 1.3	0.32 ± 0.84	0.0876
Intake of NSAIDs	15 (21.1%)	10 (27.02%)	5 (14.7%)	0.252
Intake of bisphosphonates	8 (11.3%)	6 (16.2%)	2 (5.88%)	0.264
Intake of PPI	34 (47.9%)	34 (91.9%)	0	

Data are mean ± standard deviation unless otherwise shown

Bolded values with *P* < 0.05

*ASD* adult spinal deformity*, FBB* fulcrum backward-bending, *GERD* gastroesophageal reflux disease, *LL* lumbar lordosis, *TK* thoracic kyphosis, *TLK* thoracolumbar kyphosis, *PI* pelvic incidence; *PT* pelvic tilt, *SS* sacral slope, *SVA* sagittal vertical axis, NSAIDs non-steroidal anti-inflammatory drugs, *PPI* Proton pump inhibitor

Seventy-three patients were treated with thoracolumbar corrective surgery between April 2010 and March 2014 by three board-certified spinal surgeons at a single institution; two patients missed follow-up. Therefore, 71 consecutive patients with ASD who were treated with thoracolumbar corrective surgery and followed up for a minimum of 1 year were enrolled in this study.

### Radiographic measurements

Radiographic data collection consisted of full-length lateral radiographs obtained with the patient in a freestanding posture with fingers placed on the clavicles or supine in a fulcrum backward-bending (FBB) position preoperatively. Preoperative evaluation for flexibility of the thoracolumbar spine is essential to perform the optimum surgery and includes an assessment of the necessity of osteotomy and/or range of fusion. Radiographs obtained with the supine patient in an FBB position is an effective technique to evaluate the flexibility of the thoracolumbar kyphotic curvature [14].

Recent reports indicated that the following sagittal spinopelvic parameters are directly correlated with HRQoL scores evaluated by questionnaires such as the ODI, the Scoliosis Research Society-22r, and the SF-12 [4–7]: the SVA, PT, and pelvic incidence-lumbar lordosis (PI-LL) have been known as the parameters that are most strongly correlated with disability of patients with ASD [3]. Standing full-length lateral radiographs were captured 1 year postoperatively. The following radiographic parameters were measured using a lateral view: T5−T12 TK, T10-L2 thoracolumbar kyphosis (TLK), T12−S1 LL angles, PI, PT, sacral slope (SS), and SVA. Kyphosis was expressed as a positive value, and lordosis was expressed as a negative value. Using a frontal view radiograph with the patient in the standing position, we measured the coronal Cobb angle in the thoracolumbar, lumbar and thoracic spine. Vertebral fracture was considered present if at least one of three height measurements (anterior, middle, and posterior) for one vertebra had decreased by >20% compared with the height of the nearest uncompressed vertebral body [15]. Radiographic measurements were obtained by one of the authors based on a single measurement using standardized techniques. This author has more than 10 years of experience in spinal surgery and was blinded to the Quest scores.

### Diagnosis of GERD

All patients were asked to respond to the Frequency Scale for Symptoms of GERD (FSSG) questionnaire regardless of any specific complaints. The questionnaire contains 12 questions and has been recently developed as a self-report instrument that is written in simple and easy-to-understand language. Symptom frequency was measured on the following scale: never = 0; occasionally = 1; sometimes = 2; often = 3; and always = 4. The FSSG questionnaire is appropriate for management of GERD in general practice. The results from the FSSG questionnaire have been shown to correlate strongly with endoscopic findings; thus, endoscopy is not a requirement [16].

Evaluation of GERD was conducted within 2 weeks prior to surgery and 1 year after surgical spinal correction. GERD was diagnosed by a gastroenterologist based on the patient’s response to proton pump inhibitor (PPI) medication and/or an FSSG score > 8 points [13, 16], and patients were divided into groups based on the presence or absence of GERD, that is, GERD+ and GERD−, respectively.

### Statistical analyses

All data are reported as means ± SD. Data were analyzed using a two-sided Student *t-*test and a Fisher exact test to determine significant differences. Correlations between radiographic parameters and FSSG scores were determined by Pearson’s correlation coefficient. All statistical calculations were performed with Prism version 6.0 (Graph Pad Software, La Jolla, CA). Multivariate logistic regression analyses were performed with R software, version 3.2.3, to evaluate the odds ratio (OR) with a 95% confidence interval (95% CI) for potential risk factors for GERD. For all tests, *P* < 0.05 was considered statistically significant (*P < 0.05, **P < 0.005, ***P < 0.0005, ****P < 0.0001).

## Results

### Preoperative prevalence of GERD and patient characteristics

The mean age of the patients in the study population was 70.6 ± 7.3 years, and there were 62 females and 9 males. Of the 71 patients treated surgically for ASD, 37 (52%) had a high frequency of GERD symptoms. Table 1 summarizes the preoperative baseline characteristics and spinal radiographic parameters measured in the patients in the GERD+ and GERD− groups. There were no significant between-group differences in mean age, gender balance, intake of oral NSAIDs, oral bisphosphonates or number of thoracolumbar vertebral fractures (Table 1). Of the 37 patients in the GERD+ group, 34 (91.9%) were treated with a PPI prescribed by a gastroenterologist (Table 1). In accordance with past reports [17], our data also showed that patients with a coronal Cobb angle of more than 30° had GERD symptoms more frequently (Table 1). There were no significant differences between the groups for any of the sagittal spine radiographic parameters measured in a standing position. In contrast, TK and TLK in the FBB position were significantly larger in the GERD+ group compared with the respective values in the GERD− group (Table 1).

**Fig. 1:**
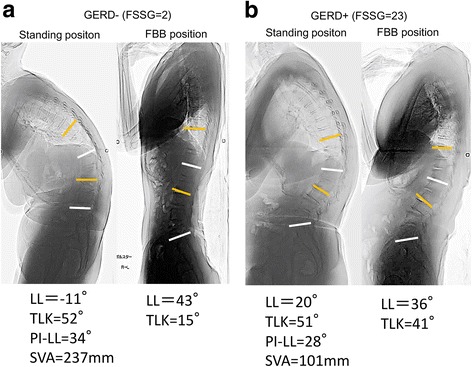
Representative cases with or without severe gastroesophageal reflux disease symptoms: Frequency Scale for Symptoms of GERD score of 2 (**a**) or 23 (**b**). The white line denotes lumbar lordosis. The yellow line denotes thoracic lumbar kyphosis **a** Lateral standing radiograph showing global sagittal malalignment due to a − 11° lumbar lordosis and a 51° thoracic lumbar kyphosis. Lateral radiograph obtained in the fulcrum backward-bending position showing thoracic lumbar kyphosis reduced to 15°. **b** Lateral standing radiograph showing a − 20° lumbar lordosis and global sagittal malalignment due to a 51° thoracic lumbar kyphosis. Lateral radiograph obtained in the fulcrum backward-bending position showing a 41° thoracic lumbar kyphosis that is not flexible.

Strong correlation coefficients were observed between TK, TLK and LL in the FBB position and in the standing position. Therefore, the multivariate logistic regression analysis was conducted twice, once with all variables excluding TK, TLK and LL in the FBB position (Table 2) and once excluding TK, TLK, and LL in the standing position (Table 3). Parameters used in the multivariate analysis were age, sex, SVA, TK, TLK, LL, PI, PT, SS and Cobb angle. The smaller LL and larger TLK curves in the FBB position and the larger coronal Cobb angle in the standing position were significantly associated with the presence of GERD (Table 3). No other variables, including sagittal parameters, were significantly correlated. Representative cases with or without severe GERD are shown in Fig. 1. Both cases had definite sagittal imbalance, including large TLK in the standing position. In contrast, only the GERD+ case had a large fixed TLK curve in the FBB position (Fig. 1b).

**Table 2:** Multivariate logistic regression analysis of risk factors for developing GERD symptoms based on radiographs in the standing position

Parameter	OR	95% CI	*P*-value
TK, standing	1.028	0.972–1.093	0.2266
TLK, standing	1.020	0.967–1.080	0.3443
LL, standing	0.998	0.939–1.063	0.9500
PI -LL	1.039	0.9477–1.167	0.4332
PT	0.982	0.882–1.067	0.6832
SS	0.939	0.832–1.034	0.2225
SVA	0.999	0.984–1.013	0.8371
Cobb angle	1.099	0.997–1.239	0.0727

*CI* confidence interval*, GERD* gastroesophageal reflux disease, *LL* lumbar lordosis, *OR* odds ratio*, TK* thoracic kyphosis, *TLK* thoracolumbar kyphosis, *PI* pelvic incidence; *PT* pelvic tilt, *SS* sacral slope, *SVA* sagittal vertical axis

**Table 3:** Multivariate logistic regression analysis of risk factors for developing GERD symptoms based on radiographs in the FBB position

Parameter	OR	95% CI	*P* value
TK, FBB	1.063	0.982–1.171	0.1546
TLK, FBB	1.192	1.065–1.397	**<0.01**
LL, FBB	0.877	0.776–0.962	**<0.05**
PI-LL	1.018	0.901–1.209	0.7701
PT	1.035	0.872–1.164	0.5870
SS	1.017	0.859–1.171	0.8111
SVA	0.996	0.978–1.015	0.6842
Cobb angle	1.217	1.051–1.495	**<0.05**

Bolded values with *P* < 0.05

*CI* confidence interval*, FBB* fulcrum backward-bending, *GERD* gastroesophageal reflux disease, *LL* lumbar lordosis, *OR* odds ratio*, TK* thoracic kyphosis, *TLK* thoracolumbar kyphosis, *PI* pelvic incidence, *PI* pelvic tilt, *SS* sacral slope, *SVA* sagittal vertical axis

### Efficacy of surgical spinal correction for GERD

**Fig. 2:**
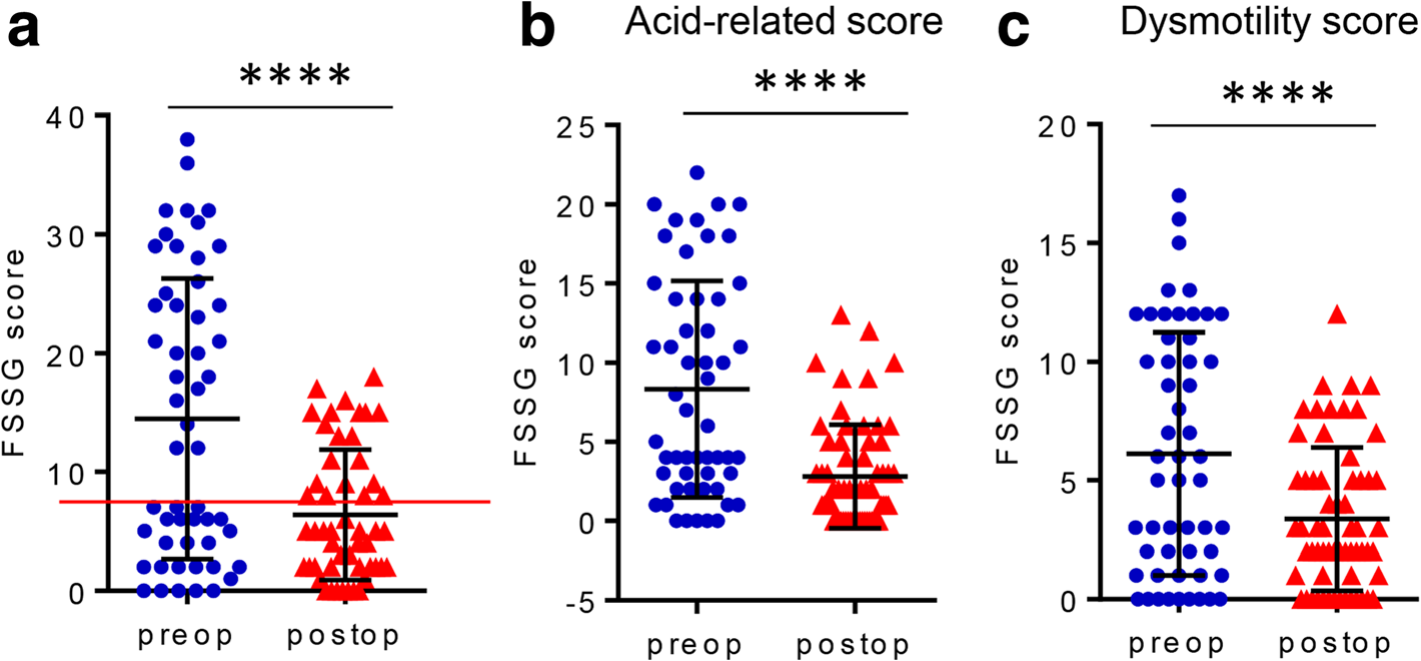
**a** Comparison of preoperative and 1-year postoperative (preop and postop) FSSG scores (****P < 0.0001). **b** Comparison of preoperative and 1-year postoperative (preop and postop) acid-related score in the FSSG scores (****P < 0.0001). **c** Comparison of preoperative and 1-year postoperative (preop and postop) dysmotility score in the FSSG scores (****P < 0.0001)

One year after surgery, the total FSSG score of ASD patients decreased significantly from 14.5 ± 11.8 (presurgery) to 6.4 ± 5.5 (postsurgery) (*P* < 0.0001) (Fig. 2a). Additionally, both the acid-related score and the dysmotility portion of the FSSG score decreased significantly (*P* < 0.0001) (Fig. 2b, c). However, of the 71 patients treated surgically for ASD, 20 (28.2%) still had GERD symptoms (FSSG score > 8 points) 1 year after surgery.

*Correlation between FSSG scores and TLK curves.*

**Fig. 3:**
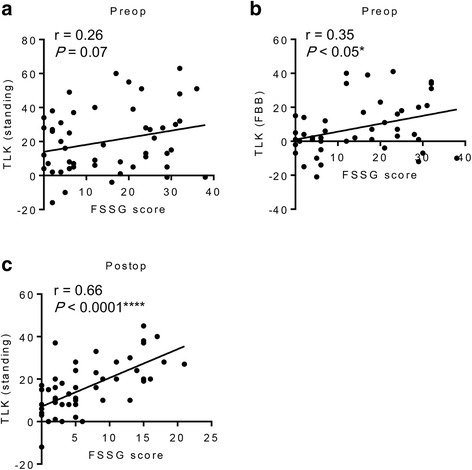
**a** Correlation between preoperative FSSG scores and preoperative standing TLK curves. **b** Correlation between preoperative FSSG scores and preoperative TLK curves in the FBB position. **c** Correlation between the 1-year postoperative FSSG scores and the postoperative standing TLK curves

The correlations between FSSG scores and TLK angles are shown in Fig. 3. No significant correlation was found between preoperative FSSG scores and preoperative standing TLK curves (Fig. 3a). In contrast, a significant positive correlation was found between preoperative FSSG scores and preoperative TLK curves in the FBB position (Fig. 3b). Surprisingly, a significant, highly positive correlation was found between FSSG scores and standing TLK 1 year after surgery (*r* = 0.66, *P* < 0.0001) (Fig. 3c).

## Discussion

Our data showed that of the 71 patients who underwent surgical treatment for ASD, 37 (52%) had a high frequency of GERD symptoms. Therefore, GERD might be one of the main complaints to evaluate when performing thoracolumbar corrective surgery. Spinal malalignment parameters may influence the risk for GERD in patients with ASD; therefore, precise information regarding these parameters should be obtained.

Age, female sex, obesity, hiatal hernia, smoking, regular consumption of alcohol, and *H. pylori* negativity have been elucidated as risk factors for GERD [8] [18–20]. In addition, the association between osteoporosis/spinal hyperkyphosis and increased prevalence of GERD has been recognized [10, 11, 13, 21]. However, these reports lacked detailed information on global spinal alignment, the location of the kyphosis, and the kyphosis angle. In accordance with past reports [17], our data also showed that scoliosis was a significant risk factor for GERD, and risk increased with a curve >30 degrees. A recent report on volunteers who attended a basic health checkup and had an evaluation of global spinal alignment indicated that the angle of the LL, sagittal balance, and back muscle strength are significant factors related to the presence of GERD [22]. In contrast to previous studies, we did not find spinal sagittal alignment based on parameters of TK, TLK, or LL measured by full-length standing spinal radiographs to be significant risk factors for GERD symptoms. This may be because our subjects were limited to patients who had serious spinal deformity. In the current study, PI-LL mismatch in the standing position also did not correlate with GERD symptoms (Table 1). The pathology underlying ASD can include a wide variety of spinal curves, such as marked global sagittal malalignment due to lumbar kyphosis or flexible or rigid TK and severe scoliosis without marked global sagittal malalignment [14]. Preoperative evaluation of the flexibility of the curvature of the spine is essential for selecting the optimal surgical strategy for patients with ASD, and it has been demonstrated that radiographs in the FBB position can be used to assess vertebral curve flexibility [14, 23, 24]. Our study showed that decreased LL and increased TLK measured in the FBB position, which indicate decompensatory sagittal malalignment, are critical risk factors for the development of GERD symptoms in patients with ASD (Fig. 1a, b).

A recent report indicated improvement of GERD in patients with spinal kyphotic deformity who underwent surgical spinal correction. However, this study did not consider the relationship between the improvement of GERD symptoms and the global spinal alignment [25]. Similar to the recent report [25], our study also showed significant improvements in FSSG scores that were noted postoperatively. In addition, we found some patients still had GERD symptoms, and postoperative fixed TLK was significantly highly correlated with FSSG scores 1 year after surgery. One possible mechanism for these results is that an increase in intra-abdominal pressure is caused by thoracolumbar kyphosis, with subsequent compression of the esophagus and stomach cranially, and this mechanism is supported by past reports [11, 22].

The limitations of this study were the small sample size and the assessment of GERD symptoms by the Quest score only, without diagnostic endoscopy or other additional tests. The sensitivity of the FSSG for diagnosis of GERD is about 70%. In addition, GERD included reflux esophagitis and non-erosive reflux esophagitis (NERD). In the current study, most patients grouped as GERD-positive were dosed with PPIs, and they may be patients with NERD or functional heartburn. Further study using endoscopic evaluation is needed to elucidate the epidemiology and pathology of GERD in ASD more precisely. Additionally, because some patients might stop the intake of PPIs postoperatively, the influence of postoperative intake of oral PPIs on FSSG score should be evaluated in a further study. However, to our knowledge, this is the first study to report the prevalence of GERD in patients with ASD, to demonstrate that a fixed thoracolumbar curve that is decreased in LL and increased in TLK in the FBB position is significantly associated with development of GERD symptoms in these patients, and to suggest that insufficient surgical correction of TLK might be a risk factor for persistent GERD symptoms. Despite it being well known that sagittal spinopelvic alignment is an important factor for optimal surgical planning, the results of the current study also indicated that management of an inflexible thoracolumbar curve is necessary to treat GERD symptoms in patients with ASD.

## Conclusions

Of the 71 patients treated surgically for ASD, 37 (52%) had a high frequency of GERD symptoms. An inflexible thoracolumbar curve with an increased TLK in the FBB position was significantly associated with GERD symptoms. Despite significant improvements in FSSG scores postoperatively, insufficient correction of TLK might be a risk factor for persistent GERD symptoms.

## Abbreviations

ASD: Adult spinal deformity
FBB: Fulcrum backward-bending
GERD: Gastroesophageal reflux disease
HRQoL: Health-related quality of life
LL: Lumber Lordosis
NERD: Non-erosive reflux esophagitis
NSAIDs: Nonsteroidal anti-inflammatory drugs
ODI: Oswestry disability index
PI: Pelvic incidence
PPI: Proton pump inhibitor
PT: Pelvic tilt
SS: Sacral slope
SVA: Sagittal vertical axis
TK: Thoracic kyphosis
TLK: Thoracolumbar kyphosis
